# Green Composites Based on Mater-Bi^®^ and *Solanum lycopersicum* Plant Waste for 3D Printing Applications

**DOI:** 10.3390/polym15020325

**Published:** 2023-01-08

**Authors:** Roberto Scaffaro, Maria Clara Citarrella, Marco Morreale

**Affiliations:** 1 Department of Engineering, University of Palermo, Viale delle Scienze, 90128 Palermo, Italy; 2 INSTM, Consortium for Materials Science and Technology, Via Giusti 9, 50125 Florence, Italy; 3 Faculty of Engineering and Architecture, Kore University of Enna, Cittadella Universitaria, 94100 Enna, Italy

**Keywords:** green composites, 3D printing, FDM, biopolymers, solanum lycopersicum

## Abstract

3D printability of green composites is currently experiencing a boost in importance and interest, envisaging a way to valorise agricultural waste, in order to obtain affordable fillers for the preparation of biodegradable polymer-based composites with reduced cost and environmental impact, without undermining processability and mechanical performance. In this work, an innovative green composite was prepared by combining a starch-based biodegradable polymer (Mater-Bi^®^, MB) and a filler obtained from the lignocellulosic waste coming from *Solanum lycopersicum* (i.e., tomato plant) harvesting. Different processing parameters and different filler amounts were investigated, and the obtained samples were subjected to rheological, morphological, and mechanical characterizations. Regarding the adopted filler amounts, processability was found to be good, with adequate dispersion of the filler in the matrix. Mechanical performance was satisfactory, and it was found that this is significantly affected by specific process parameters such as the raster angle. The mechanical properties were compared to those predictable from the Halpin–Tsai model, finding that the prepared systems exceed the expected values.

## 1. Introduction

Over the last few decades, increasing attention has been focused on the ways to improve the cost-effectiveness of the production of polymer-related items, by possibly replacing part of the polymer needed to manufacture a certain product with waste materials and/or by-products coming from other industrial, or agricultural, operations; at the same time, the need and interest in reducing the environmental impact related to the entire life cycle of polymer-based goods have grown exponentially, suggesting to replace (at least) part of the polymer itself with materials coming from renewable sources and/or biodegradable [1]. Furthermore, it is obvious that a more significant reduction in the environmental impacts requires replacing traditional polymers (coming from non-renewable sources) with bio-based and, preferably, also biodegradable polymers. Among the waste materials which can be conveniently used as fillers for polymers systems, agricultural, marine, or industrial wastes from wood processing are particularly attractive; at the same time, it is important to use biodegradable polymers in order to reduce the environmental pollution related to plastics [1,2,3,4,5,6,7,8] and to focus on obtaining a satisfactory mechanical behaviour [1,2,3,4,5].

In this background, the biopolymers which are more typically used in the preparation of green composites are poly (lactic acid) (PLA), polybutylene adipate terephthalate (PBAT), polycaprolactone (PCL), cellulose and starch-based polymers [2,6]. For instance, Mater-Bi^®^ (MB) is a family of commercial starch-based biopolymers that have been finding interesting applications in many fields, thanks to satisfactory mechanical properties, good processability, adequate thermal stability, biodegradability/compostability and suitability to be reinforced with natural-organic fillers, as already reported by many papers [4,5,7]. It is important to observe that the addition of a natural-organic filler to such polymer matrices was found to improve the biodegradability [6,7] and, often, to improve the mechanical behaviour [2,7,9,10,11]: therefore, plant-based biomasses should be investigated for their actual potential in achieving both of such fundamental targets and they should preferably hold the prerequisite of being easily available, cheap, and widely present on the territory.

The Mediterranean area offers a wide variety of plant species (or, in general, lignocellulosic sources), coming from either the agricultural or the marine environment, that can effectively find applications in the preparation of polymer-based biocomposites. For instance, these can include Opuntia Ficus Indica (OFI), Posidonia Oceanica (PO) and Hedysarum coronarium (HC). OFI has already been studied in combination with PLA, to produce green composites via the compression moulding technique [9]. PO and in particular PO leaves (POL) have been investigated in several studies, focused on the structure–properties relationships; finding that the mechanical behaviour can be enhanced and, quite interestingly, that the degradability can be accelerated by the presence of POL [10,12,13]. HC is very abundant in the Mediterranean area and is known for applications in the agri-food sector [14,15] but has been recently investigated also regarding the formulation and preparation of green composites [16,17].

However, the formulation and preparation of innovative and effective green composites cannot be based only on the choice of the polymer matrix and the filler, but it must also consider the choice and setup of the optimal processing technique. To this point, it should be observed that thermoplastic-based green composites are usually produced by compression moulding, extrusion, or injection moulding [18]; on the other hand, the continuous development of new and more versatile production solutions, has led to a significant interest in fused deposition modelling (FDM), a technique (often referred to as “3D printing”) which is now known for its great versatility: it allows obtaining elaborated geometries while still granting significant reductions in time and costs, and thus it is already one of the most promising also with concern to green composites [19,20,21,22,23].

More specifically, there are some recent works where lignocellulosic wastes have been used as fillers for green composites and investigated for actual suitability to FDM manufacturing. HC was combined with Mater-Bi^®^ (MB) [16] or PLA [17] and the green composites were prepared via two different routes, i.e., compression moulding (CM) or FDM. It was found [16] that FDM could be preferable up to 10% HC content, leading to better mechanical properties (in particular, with regard to the elastic modulus) in comparison to CM, likely due to rectilinear infill and fibres orientation; furthermore, it was possible to get more dense structures than by CM [17], obtaining quite significant improvements of the mechanical properties (especially flexural ones) in comparison to the neat polymer. OFI and/or POL were investigated in combination with PLA and processed via FDM, finding that it was possible to replace up to 20% of the polymer matrix [24], with final samples characterized by good mechanical properties and satisfactory filler dispersion as well as filler–matrix adhesion, with very interesting potential applications in the release of fertilizers [25].

As pointed out several times over this brief bibliographic overview, one of the main goals related to the development and use of green composites depends on the utilization of natural-organic wastes, coming from flora (both terrestrial and marine) or fauna [26]. From this point of view, one interesting source may come from *Solanum lycopersicum*, i.e., tomato plant. This plant, widely grown in temperate zones across the world, and also in greenhouses, is one of the most important for its edible purpose. Tomato production in 2020 was led by China with almost 65 million tons, followed by India, Turkey, the United States and Egypt [27]. During the production and transportation stages, several wastes are typically produced, accounting for an estimated 10–15% of the total volume and are commonly used for compost or animal feed [28]. These wastes basically consist of skin, seeds, and tomato pomace (a by-product of tomato processing, based on peel, seeds and small amounts of pulp) and many investigations are recently focused on how to exploit them for higher-value purposes, such as extraction of lycopene, carotenoids, bases for biofuels, etc. [28,29]. However, much less attention is focused on the lignocellulosic wastes coming from the plants after extirpation of the fruits. Such lignocellulosic wastes are usually driven to incineration or, when discarded on the ground, they can represent a significant hazard, since they may contribute to feed fires and related events. It would be therefore preferable to find alternative solutions for such wastes, and their proper incorporation into green composites may be an optimal way. To our best knowledge, there is no evidence in the literature about systematic studies on green composites based on biodegradable polymers (in particular, from the Mater-Bi^®^ family) and fillers obtained from *Solanum lycopersicum,* let alone via a more innovative technique such as FDM.

In this paper, therefore, we prepared composites based on a Mater-Bi^®^ polymer and wastes coming from *Solanum lycopersicum*, processing them via FDM, in order to explore the actual suitability to 3D-printing applications. The obtained samples were characterized from the rheological, mechanical, and morphological points of view.

## 2. Materials and Methods

### 2.1. Materials

The biopolymeric matrix used to prepare the green composites was a sample of Mater-Bi^®^ EF51L (MB) supplied by Novamont SpA (Novara, Italy), a polymer based on blends of aromatic and aliphatic biodegradable co-polyesters with proprietary composition. In order to avoid hydrolytic chain scissions during the melt processing, neat MB and MB-based composites were vacuum-dried overnight at 60 °C before each process.

*Solanum lycopersicum* plant waste (SL) used in this study was kindly supplied by a local farm (Sicily, Italy) The plants were mowed after tomato harvesting. In this study, the whole plant was ground as received in order to optimize production time and costs. More in detail, the obtained plant wastes were washed and dried in a vacuum oven (NSV9035, ISCO, Milan, Italy) at T = 40 °C for 3 days, and finally ground using a laboratory grinder (Retsch, Germany).

SL dried stem showed a Young’s modulus of 404 MPa. The flour, obtained by grinding the whole plant as described above, displayed an average density of 1.87 g/cm^3^. It was further vacuum-dried, overnight at 40 °C, prior to the melt mixing process in order to reduce potential MB chain scission phenomena during processing.

### 2.2. Composites Preparations

Firstly, the dried SL plant was ground for 3 min in a grinder (Retsch, Germany). The resulting powder was then sieved to obtain particles of a size suitable for the 3D printer (Next Generation, Sharebot, Nibionno, Italy), which, therefore, do not lead to obstructions in the nozzle. To this aim, and based on previous studies [11,20], the sieving fraction under 150 μm was selected. Prior to processing, the obtained SL flours and MB pellets were dried overnight in a vacuum oven (NSV9035, ISCO, Milan, Italy) at 40 °C and 60 °C, respectively.

In order to obtain a homogeneous dispersion of the filler, according to previous studies, the filler amounts chosen to prepare the MB-based biocomposites were 5, 10, 15 wt%. All of the composites (namely MB/SL5, MB/SL10, MB/SL15) and neat MB, for comparison, were prepared by melt compounding in an internal mixer (Plasticorder, Brabender, Duisburg, Germany; T = 160 °C, rotor speed = 64 rpm, t = 5 min).

The obtained materials were then ground into pellets and processed in a Polylab single-screw extruder (Haake Technik GmbH, Vreden, Germany; L/D = 25; D = 19.05 mm), operating at 40 rpm screw speed and 130–140–150–160 °C temperature profile. The extrudates were drawn with the help of a conveyor belt system (take-up speed = 5.5 m/min), to obtain filaments with a diameter suitable to the printer (1.75 mm).

The samples obtained for fused deposition modelling (FDM) were first designed with the help of CAD Solid Edge 2019^®^ software (Plano, TX, USA), and the STL files produced were elaborated on Simplify3D^®^ software (Cincinnati, OH, USA) to obtain the related gcode files. For each formulation, 60 mm × 10 mm × 1 mm samples were printed using a Sharebot Next Generation (Nibionno, Italy) 3D printer. FDM operating parameters are reported in Table 1. Nozzle temperature was chosen after some trials, aiming to avoid nozzle obstructions and to obtain good printability performance. The other parameters were chosen based on the scientific literature [16,17,24,25,26]. In particular, a 100% infill rate and a rectilinear infill pattern with a 0° or ±45° raster angle were chosen in order to evaluate its influence on the tensile properties of the composites; 45 mm/s printing speed was chosen to maximize the production rate without compromising the mechanical performance.

Sample formulations and sample codes are reported in Table 2.

### 2.3. Characterizations

#### Rheological Characterization

Rheological properties of the samples were analysed, using a rotational rheometer (ARES-G2, TA Instruments, New Castle, DE, USA) equipped with a 25 mm parallel-plate geometry. All the tests were performed at 160 °C, in frequency sweep mode in the range 1–100 rad/s, by imposing a constant stress of 1 Pa.

### 2.4. Morphological Analysis

The morphology of SL powder, composites filaments and FDM samples was observed by using a scanning electron microscope (Phenom ProX, Phenom-World, Eindhoven, The Netherlands) with an optical magnification range of 20–135×, electron magnification range of 80–1.3 × 10^5^, maximal digital zoom of 12×, and acceleration voltages of 15 kV. The microscope is equipped with a temperature controlled (25 °C) sample holder. The samples were fixed on an aluminium stub (pin stub 25 mm, Phenom-World, Eindhoven, The Netherlands) using a glued carbon tape.

### 2.5. Mechanical Characterization

The mechanical behaviour of SL plant, composites filaments and FDM-printed samples was investigated by tensile tests, carried out using a laboratory dynamometer (mod.3365, Instron, Norwood, MA, USA) equipped with a 1 kN load cell. The tests were performed on rectangular-shaped specimens (60 mm × 10 mm) according to ASTM D638. In particular, the measurements were performed by using a double crosshead speed: 1 mm min^−1^ for 2 min and 50 mm min^−1^ until fracture occurred. The grip distance was 30 mm, whereas the sample thickness was measured before each test. Eight specimens were tested for each sample, and the results for elastic modulus (E), tensile strength (TS) and elongation at break (EB) have been reported as the average values ± standard deviations.

### 2.6. X-ray Diffraction

X-ray diffraction patterns were collected by using a RIGAKU diffractometer (D-MAX 25600 HK, Rigaku, Tokyo, Japan). All diffraction patterns were obtained in the 2θ range from 5° to 80° by means of copper Kα radiation (λ = 1.54 Å) with the following setup conditions: tube voltage and current of 40 kV and 30 mA, respectively, scan speed of 4°/min with a sampling of 0.004°.

### 2.7. Differential Scanning Calorimetry Analysis

Differential scanning calorimetry (DSC) analysis was carried out on a Chip-DSC 10 (Linseis Messgeraete GmbH, Selb, Germany) by heating the samples to 200 °C at a heating rate of 40 °C/min.

### 2.8. Density Measurements

Density measurements were performed by a Thermo Pycnomatic Helium Pycnometer (Pycnomatic ATC, Thermofisher, Waltham, MA, USA), using 99.99% pure helium. Measures were repeated at least six times for each sample, at 25 °C.

### 2.9. Theoretical Modelling

The outcomes of the tensile tests were compared with those predicted by the Halpin–Tsai model, which allows esteeming the modulus of composites once are known volume fractions and elastic moduli of the starting components, and the filler aspect ratio. According to the Halpin–Tsai model, for composites reinforced with fibres randomly oriented, the composite modulus *E_C,HT_* is determined by the following equation:

(1) $$E_{C,\;HT}=\frac{3}{8}E_{L}+\frac{5}{8}E_{T}$$

where $E_{L}\;$ and $E_{T}$ are, respectively, the longitudinal and transverse moduli of the composite.

In this case, $E_{L}$ and $E_{T}$ are given by:

$$E_{L}=E_{m}\left[\frac{1+\left(2l/d\right)\eta_{L}\upsilon_{f}}{1-\eta_{L}\upsilon_{f}}\right]\;\;\;\;\;\;\;\;\;\;\;\;\;\;\;\;\;\;\;\;\;\;\;\;\;\;\;E_{T}=E_{m}\left[\frac{1+2\eta_{T}\upsilon_{f}}{1-\eta_{L}\upsilon_{f}}\right]\;\;\;\;\;\;$$

where *υ_f_* and *υ_m_* are the volume fractions of EE fillers and MB, respectively, l/d is the aspect ratio of the fillers while $\eta_{L}$ and $\eta_{T}$ are constants given by:

$$\eta_{L}=\frac{\left(E_{f}/E_{m}\right)-1}{\left(E_{f}/E_{m}\right)+\left(2l/d\right)}\;\;\;\;\;\;\;\;\;\;\;\;\;\;\;\;\;\;\;\;\;\;\;\;\;\eta_{T}=\frac{\left(E_{f}/E_{m}\right)-1}{\left(E_{f}/E_{m}\right)+2}\;\;\;\;\;\;\;\;$$

where, $E_{f}$ and $E_{m}$ are, respectively, the Young’s moduli of filler and MB.

Volume fractions are determined from the weight fractions and the densities of each component (i.e., SL and MB) measured experimentally by a helium pycnometer.

## 3. Results and Discussion

The samples loaded at 5% (MB/SL5), 10% (MB/SL10) and 25% (MB/SL25) filler where properly extruded into the related filaments, to be subjected to FDM thereafter.

Filament printability (i.e., processability in FDM mode) is directly correlated to the morphological properties. More in detail, not only the diameter of the filament must be suitable for the specific 3D printer used, but its surface must be as even and homogeneous as possible [26,30]. In addition, printability depends also on the rheological and mechanical properties of the filaments, which were thus investigated as well. The obtained results are discussed in the following.

### 3.1. SL Powder and Filament Characterization

**Figure 1:**
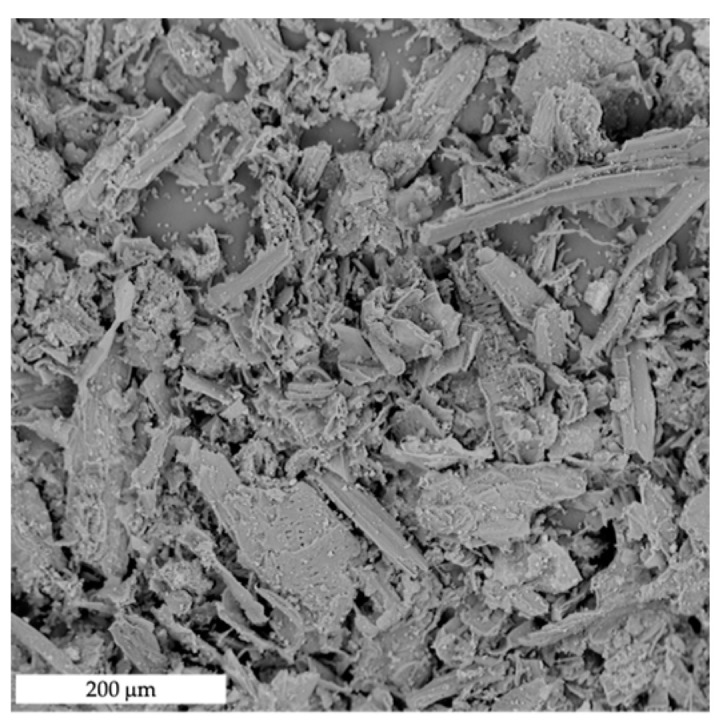
SEM images of SL powder.

**Figure 2:**
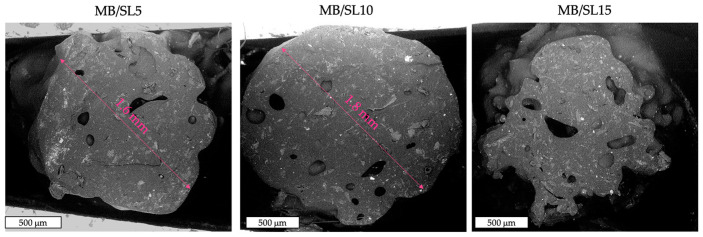
SEM images of MB/SL5, MB/SL10 and MB/SL15 filaments.

Morphological characterization was carried out first. The main results are shown in the SEM micrographs reported in Figure 1 for SL powder and in Figure 2 for MB/SL5, MB/SL10 and MB/SL15, respectively. From the SEM micrograph of the powder (Figure 1), it is possible to notice that SL powder contains elements with different morphology, reasonably belonging to different parts of the plant: stem and leaf.

From the samples’ cross-section micrographs (Figure 2), it can be observed that the SL particles are homogeneously dispersed in the MB matrix, only a few voids are present, and the general adhesion between the matrix and the particles is good. Furthermore, the diameters of the MB/SL5 and the MB/SL10 filaments are even and homogeneous, in the range 1.6–1.8 mm (respectively) which is suitable for the actual printer used. On the other hand, the MB/SL15 filaments showed uneven diameters.

Rheological measurements were performed on specimens obtained from the filaments, in order to evaluate the actual processability for FDM purposes.

**Figure 3:**
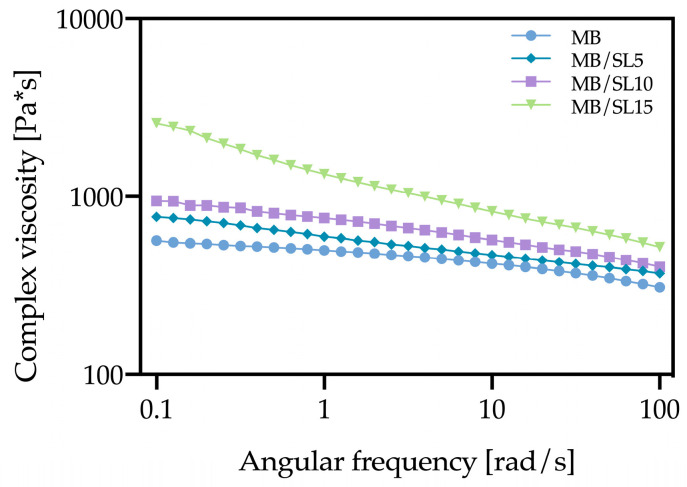
Rheological curves of MB/SL5, MB/SL10 and MB/SL15 filaments.

Figure 3 reports the rheological values of MB and the composite filaments, on increasing the filler content.

As predictable, MB shows a clear non-Newtonian behaviour. The addition of 5% SL leads to an increase of viscosity over the entire frequency range, as well as a more marked non-Newtonian behaviour. This tendency further increases by adding 10% SL. When 15% SL is added to the MB matrix, there is a much more drastic increase in the viscosity and the onset of yield stress phenomena. Such results suggest that only the rheological behaviour of MB/SL5 and MB/SL10 appears compatible with the 3D printing process, whereas MB/SL15 may be not adequately printable, due to the excessively high viscosity which may lead to nozzle clogging during the process [26].

**Figure 4:**
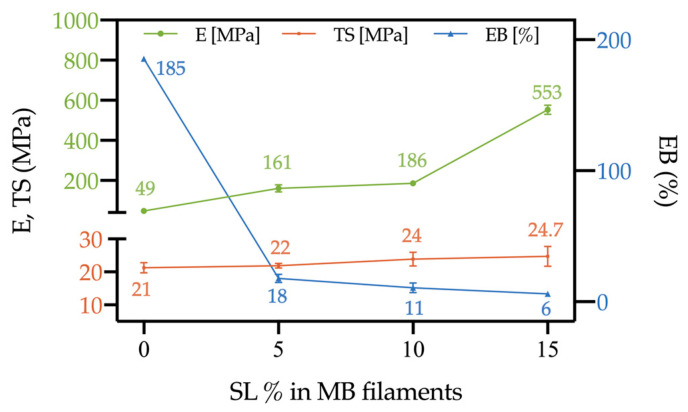
Tensile properties of MB/SL5, MB/SL10 and MB/SL15 filaments as a function of the SL amount.

Anyway, optimal printability depends also on the tensile properties of the filaments. Figure 4 reports the values of elastic modulus (E), tensile strength (TS) and elongation at break (EB), on increasing the SL content. It can be noticed that the filaments become stiffer on increasing the SL content, although the effect is much more significant only in the case of MB/SL15; the tensile strength is similar to that of the neat MB, or even higher, and this is a satisfactory result since it suggests that the filament should not undergo rupture too easily, during the process; on the other hand, the deformability drops even at just 5% SL content.

**Figure 5:**
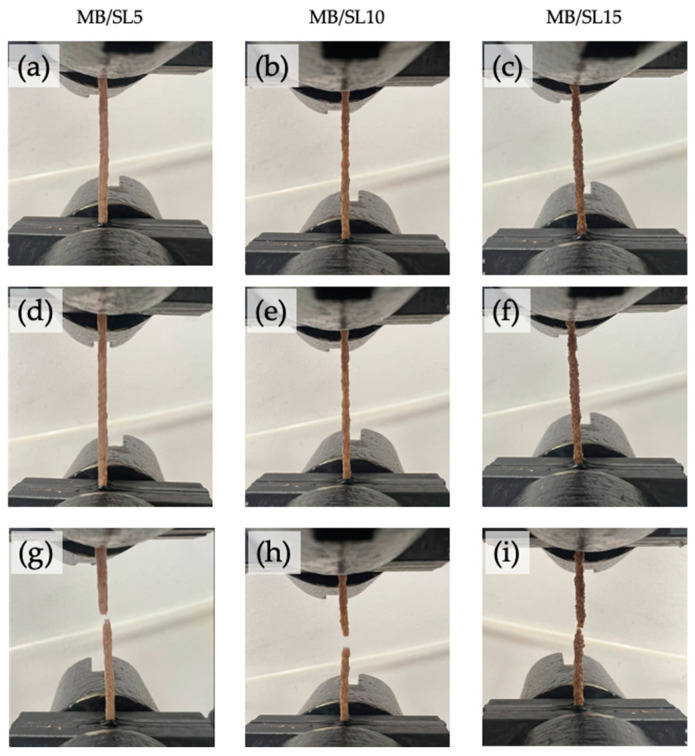
Photos of MB/SL5, MB/SL10 and MB/SL15 filaments before (**a**–**c**, respectively), during (**d**–**f**, respectively) and after (**g**–**i**, respectively) tensile test.

In Figure 5, photos of MB/SL5, MB/SL10 and MB/SL15 filaments before (Figure 5a–c, respectively), during (Figure 5d–f, respectively) and after (Figure 5g–i, respectively) tensile test are reported. MB/SL5 and MB/SL10 filaments present a homogenous shape, and their fracture occurs a few seconds after the 50 mm min^−1^ speed was applied. On the other hand, the MB/SL15 filament presents an irregular shape due to the high content of filler. In this latter case, the fracture occurred instantaneously when the 50 mm min^−1^ speed was applied.

**Figure 6:**
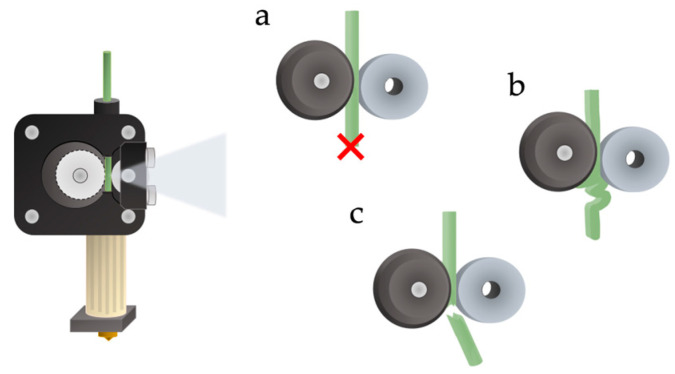
Different behaviours of the filament upon entering the melting chamber, at different viscoelastic and mechanical properties.

The results of the rheological and mechanical tests allow drawing some general considerations, propaedeutic for the FDM stage, since viscoelasticity and tensile strength measurements help to predict problems in printability and possible printing errors [31] In particular, too high viscosities are not suitable for the process, since the low deformability can lead to filament blocking at the nozzle of the 3D printer, and subsequent clogging and rupture (Figure 6a). On the other hand, if the filament is too soft (high decline of viscosity at low temperatures), it tends to flow too easily while not pulling correctly, resulting in nozzle clogging (Figure 6b); furthermore, if it is too brittle, it will break (Figure 6c) [31].

These considerations, therefore, suggest that MB/SL5 and MB/SL10 should be easily printable and without significant defects in the obtained samples (on the other hand, neat MB may lead to some uncertainty due to the relatively high deformability), while problems may arise with MB/SL15.

### 3.2. Printing of the Composites Filaments

Actual 3D printing was then carried out. As expected, based on the previous considerations, neat MB as well as MB/SL5 and MB/SL10 were easily processed, while the filament containing 15% SL showed to be not printable since the high viscosity caused obstruction of the nozzle and the filament broke easily.

The samples were printed with both 0° and 45° raster angles, in order to evaluate the printability and the effect of the angle on the mechanical properties of the obtained 3D specimens.

### 3.3. Characterizations of 3D Printed Samples

First, the 3D-printed samples were subjected to morphological analysis on cryofractured surfaces.

**Figure 7:**
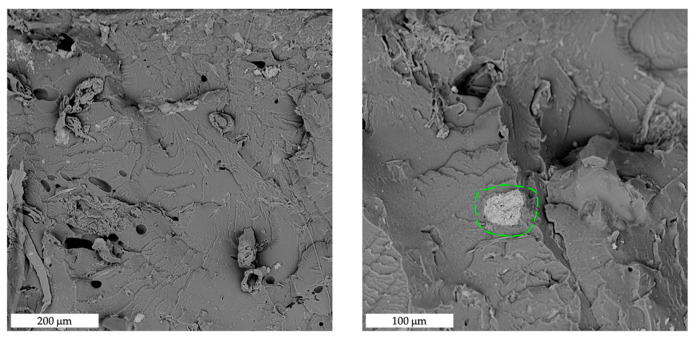
SEM images of fracture surfaces of MB/SL10 0° samples at increasing magnification (from left to right). The green circle highlights the good adhesion between the matrix and the filler.

Figure 7 shows SEM images of fractured surfaces, at increasing magnification from left to right, of MB/SL10, 0° raster samples. In general, it can be stated that filler dispersion and adhesion are good, as clearly visible from the filler particle circled in green (right), where no significant voids can be found at the filler–matrix interface.

**Figure 8:**
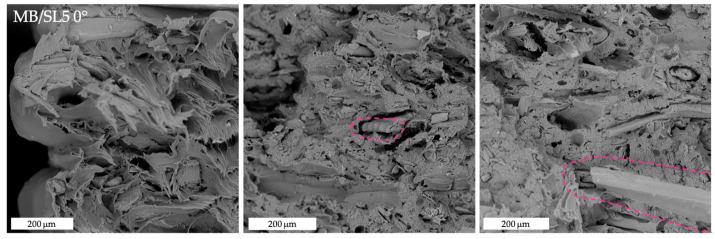
SEM images of tensile fracture surfaces of MB/SL5 0° samples. The pink circles highlight fibre pull-out and debonding phenomena.

**Figure 9:**
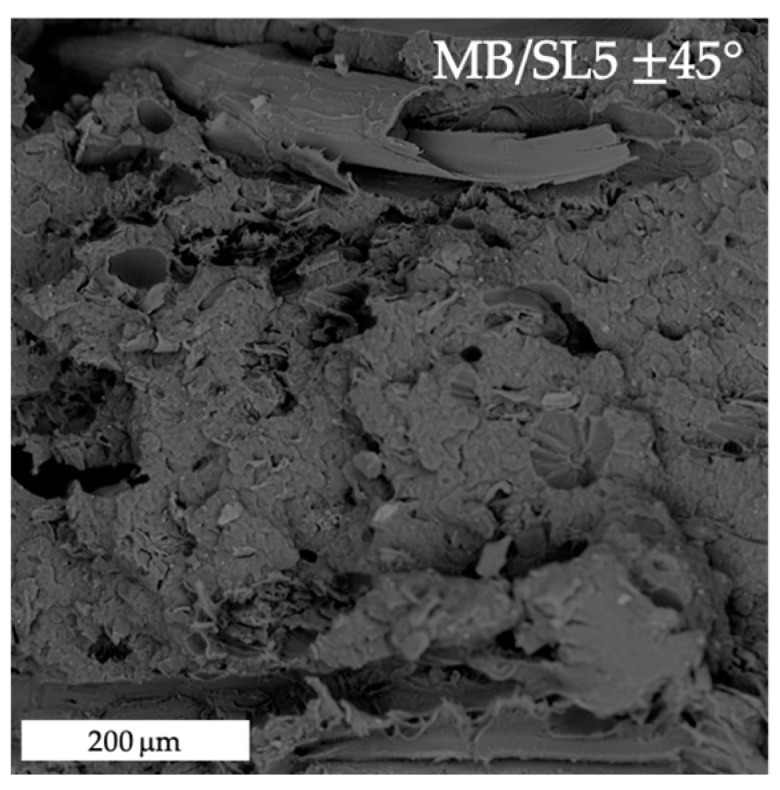
SEM image of tensile fracture surface of MB/SL5 ± 45° sample.

**Figure 10:**
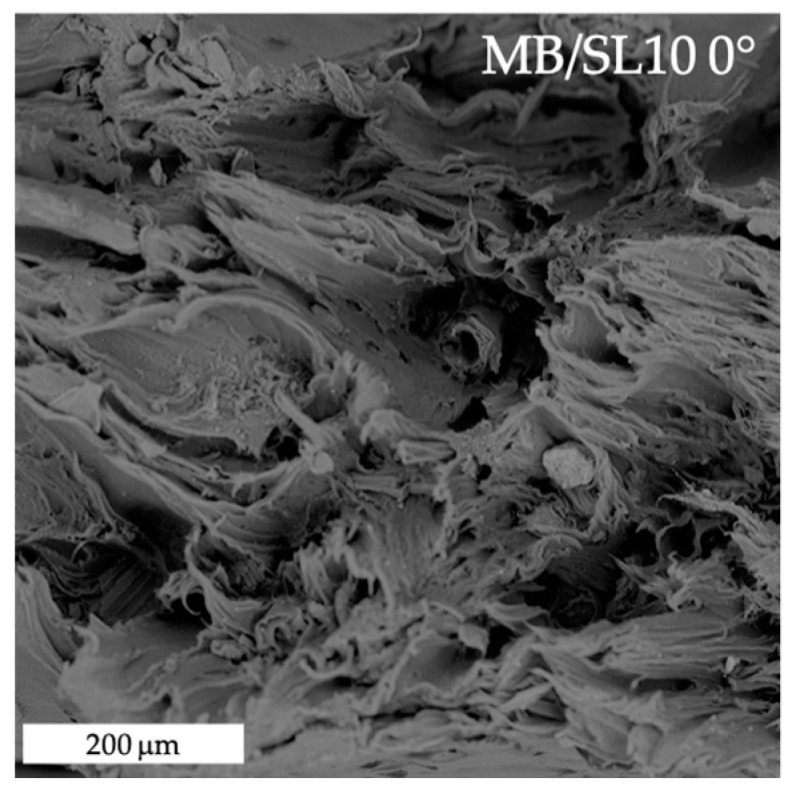
SEM image of tensile fracture surface of MB/SL10 0° sample.

**Figure 11:**
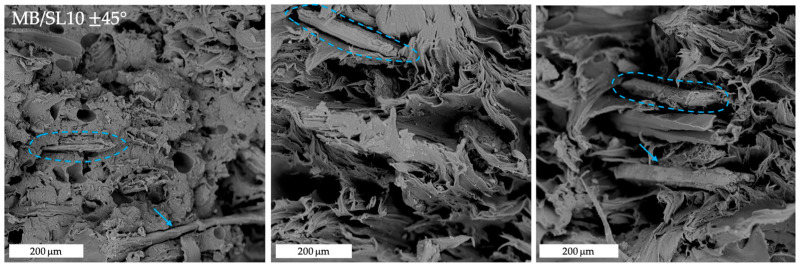
SEM images of tensile fracture surfaces of MB/SL10 ± 45° samples. The blue circles and arrows highlight fibre pull-out and debonding phenomena.

Figure 8, Figure 9, Figure 10 and Figure 11 show the fracture surfaces after tensile tests of the composite samples. Overall, it may be stated that some fibre pull-out and debonding phenomena are more visible in the SL5 rather than in the SL10 samples and, especially, in 45° samples (Figure 11) as opposed to 0° ones (Figure 10).

**Figure 12:**
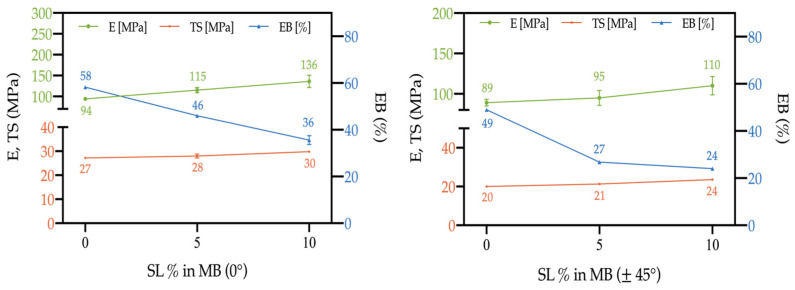
Elastic modulus (E), tensile strength (TS) and elongation at break (EB) of 3D-printed samples as a function of the filler content; raster angle = 0° (left) and raster angle = 45° (right).

The actual tensile properties of the 3D-printed samples are shown in Figure 12, in the case of raster angle = 0° (left) and raster angle = 45° (right). It can be observed that, in both cases, the elastic modulus and the tensile strength increase on increasing the filler content, while the deformability decreases. However, such a decrease is significantly less marked in the case of raster angle = 0°, and the overall results of all the tensile properties are better, with excellent reproducibility. This confirms the first indications from the morphological analysis, which could allow supposing higher breaking resistance of the 0° samples, in comparison to the 45° ones.

**Figure 13:**
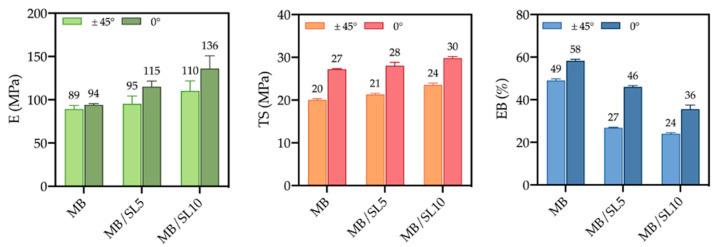
Elastic modulus (E), tensile strength (TS) and elongation at break (EB) of 3D-printed samples with different filler content and raster angle.

Such evidence can be further deduced from Figure 13. The 0° raster angle during printing definitively optimizes the tensile properties, confirming data from the literature, obtained on similar systems, where 0° raster angle usually optimizes tensile properties, whereas 45° leads to optimization of flexural and impact properties [10,26,32,33].

### 3.4. XRD and DSC Characterizations

**Figure 14:**
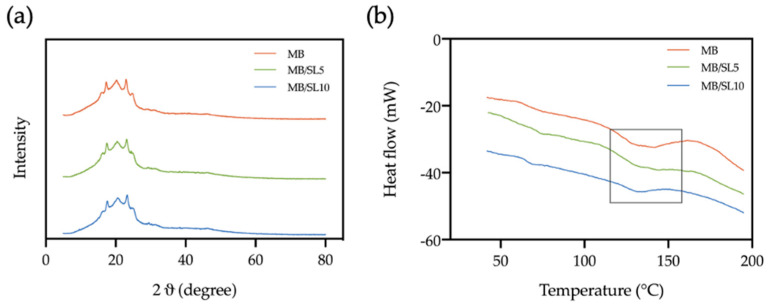
XRD spectra (**a**) and DSC analysis (**b**) of neat MB and MB/SL−printed composites.

In order to verify if the addition of SL filler leads to some crystallinity variation in the polymeric matrix, XRD and DSC analysis were performed on neat MB and MB/SL printed composites and the related outcomes are reported in Figure 14a,b, respectively. No differences can be noted in XRD curves (Figure 14a) when 5 or 10% of SL is added to the polymeric matrix. Moreover, the addition of SL powder to MB does not lead to any significant change in its melting temperature or melting enthalpy (see Figure 14b and Table 3).

These outcomes confirm that the increase in the tensile property of SL composites, if compared to the pure matrix, can be effectively attributed to the reinforcing effect given by the filler.

### 3.5. Halpin–Tsai Model

**Figure 15:**
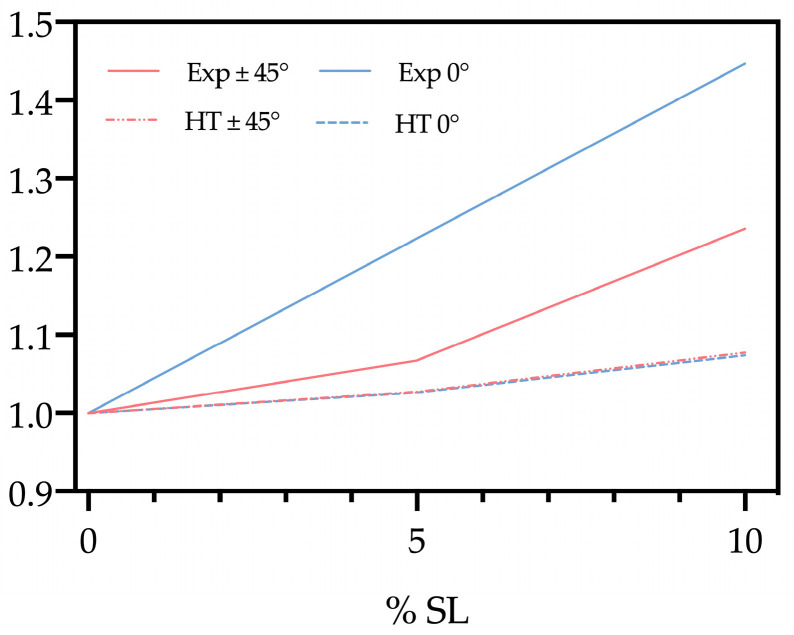
Ratio between elastic modulus of the composite and the polymer matrix, as a function of the SL content, according to the Halpin–Tsai model (HT) and the experimental results (Exp).

Figure 15 shows the trends of Ec/Em (ratio between the elastic modulus of the composite and that of the matrix) on increasing the SL content, both from the experimental (Exp) results (at 0- and 45-degree raster angles) and the theoretical trend calculated according to the Halpin–Tsai model (HT). This semiempirical model allows assessing the composite modulus (Ec), once five parameters are known, i.e., the elastic modulus of matrix (Em) and filler (Ef), their volume fractions and the filler aspect ratio [34].

The trends clearly outline that the model significantly underestimates the values of Ec, especially at higher filler contents. This may be due to the filler particles coming from different parts (wastes) of the tomato plant, thus presenting some natural differences in terms of morphology and/or mechanical properties. An additional likely explanation may involve the capability of the polymer matrix to, at least partially, enter the void channels of SL particles, as presumable on the basis of the SEM images and of the results from our previous studies on similar (i.e., biodegradable polymer/natural-organic plant waste filler) systems [9].

## 4. Conclusions

In this paper, composites based on a Mater-Bi^®^ polymer and wastes coming from *Solanum lycopersicum* were prepared and processed via FDM, in order to explore the actual suitability to 3D-printing applications. Different processing parameters and different filler amounts were investigated, and the obtained samples were characterized from the rheological, mechanical and morphological point of view. The adopted processing parameters allowed optimal processability up to 10% filler content, with satisfactory dispersion of the filler in the matrix; the same holds for the interfacial adhesion. Mechanical characterization showed that the tensile strength was kept or even improved upon increasing the filler content, the elastic modulus was enhanced and only a “physiological” reduction in the elongation at break was found; moreover, the processing parameters and, in particular, the raster angle significantly affected the tensile resistance, with 0° being preferable to ±45°. The experimental mechanical behaviour was compared to the Halpin–Tsai model, finding positive deviations for the prepared systems. Moreover, the addition of a natural waste would allow lowering the final cost of the product. Actually, the cost of *Solanum Lycopersicum* plant waste used in this work is virtually zero, since these are residues from the harvesting, and they would not find many significantly valuable alternative uses. Overall, these green composites have great potential for the development of sustainable bio-based materials aimed at several applications.

## Figures and Tables

**Table 1:** FDM process parameters.

FDM Operating Parameter	Value
Nozzle temperature	160 °C
Bed temperature	60 °C
Infill rate	100%
Infill pattern	Rectilinear
Raster angle	0° or ±45°
Layer thickness	0.1 mm
Extrusion width	0.4 mm
Printing speed	50 mm/s
Perimeter shells	1
Sample Orientation	flat

**Table 2:** Formulation of investigated samples.

Sample Code	MB Content (wt%)	SL Content (wt%)	SL Mesh Size (μm)	Raster Angle
MB 0°	100	0	-	0°
MB/SL5 0°	95	5	<150	0°
MB/SL10 0°	90	10	<150	0°
MB/SL15 0°	85	15	<150	0°
MB 45°	100	0	-	±45°
MB/SL5 45°	95	5	<150	±45°
MB/SL10 45°	90	10	<150	±45°
MB/SL15 45°	85	15	<150	±45°

**Table 3:** Melting temperature and melting enthalpy of 3D-printed samples obtained by DSC analysis.

Sample	Weight (mg)	Melting Temperature (°C)	Melting Enthalpy (mJ/mg)
MB	10.9	132.3	15.8
MB/SL5	8.8	132.7	16.3
MB/SL10	3.3	131.7	15.9

## Data Availability

The rough/processed data that support our study are available from the corresponding author on reasonable request.
